# Clinical Presentation and Management of a Dinutuximab Beta Extravasation in a Patient with Neuroblastoma

**DOI:** 10.3390/children8020091

**Published:** 2021-01-29

**Authors:** Michael Launspach, Marita Seif, Theresa M. Thole, Patrick Jesse, Joachim Schulz, Johannes H. Schulte, Susan Bischoff, Angelika Eggert, Hedwig E. Deubzer

**Affiliations:** 1 Department of Pediatric Hematology and Oncology, Charité—Universitätsmedizin Berlin, 13353 Berlin, Germany; michael.launspach@charite.de (M.L.); theresa.thole@charite.de (T.M.T.); patrick.jesse@charite.de (P.J.); joachim.schulz@charite.de (J.S.); johannes.schulte@charite.de (J.H.S.); angelika.eggert@charite.de (A.E.); 2 Berliner Institut für Gesundheitsforschung (BIH), 10178 Berlin, Germany; 3 Pharmacy, Charité—Universitätsmedizin Berlin, 13353 Berlin, Germany; marita.seif@charite.de (M.S.); susan.bischoff@charite.de (S.B.); 4 German Cancer Consortium (DKTK), Partner Site Berlin, 10117 Berlin, Germany; 5 German Cancer Research Center Heidelberg (DKFZ), 69120 Heidelberg, Germany; 6 Experimental and Clinical Research Center (ECRC) of the Charité—Universitätsmedizin Berlin and the Max-Delbrück-Center for Molecular Medicine (MDC) in the Helmholtz Association, 13125 Berlin, Germany

**Keywords:** cancer, immunotherapy, monoclonal antibody, soft tissue, inflammation

## Abstract

Extravasation can present serious accidental complication of intravenous drug application. While monoclonal antibodies do not show the necrotic potential of cytotoxic chemotherapy drugs, considerable inflammatory toxicity can occur, necessitating standardized operating procedures for the management of their extravasation. Here, we report the clinical course and management of dinutuximab beta extravasation in a 3-year-old child. Dinutuximab beta is a chimeric monoclonal antibody targeting the GD2 disialoganglioside on the surface of neuroblastoma cells that has in recent years gained significant importance in the treatment of high-risk neuroblastoma, now contributing to both first- and second-line therapy protocols. The dinutuximab beta extravasation reported here occurred when the patient received the antibody cycle as a continuous infusion over a 10-day period after haploidentical stem cell transplantation for relapsed high-risk neuroblastoma. The extravasated dinutuximab beta caused local pain, swelling, and hyperemia accompanied by fever and an overall deterioration in the general condition. Laboratory diagnostics demonstrated an increase in C-reactive protein level and total white blood cell count. Clinical complication management consisted of intravenous fluid therapy, local dabbing with dimethyl sulfoxide (DMSO), analgesia with dipyrone, as well as application of intravenous antibiotics to prevent bacterial superinfection in the severely immunocompromised host. The patient considerably improved after six days with this treatment regimen and fully recovered by day 20.

## 1. Introduction

Dinutuximab beta is a chimeric human/mouse monoclonal IgG_1_ antibody produced in the CHO (Chinese hamster ovary) mammalian cell line using recombinant DNA technology. It recognizes the GD2 disialoganglioside, which has limited expression in normal tissues but is highly expressed across several tumor entities including neuroblastoma [1]. Among normal tissues, it is found highly expressed in the central and peripheral nervous system including peripheral pain fibers [1].

Dinutuximab beta binding to GD2 mediates neuroblastoma cell killing through complement activation and effector cell-mediated cytotoxicity by the activation of Fc receptors on granulocytes and mononuclear cells [1,2]. Based on its chemical structure, it is reasoned that dinutuximab beta is eliminated after proteolytic decomposition [2]. Adverse effects of dinutuximab beta include pain, hypersensitivity reactions, and capillary leak syndrome [1,2].

Neuroblastoma is the most common extracranial solid tumor of infancy and childhood and is characterized by a diverse biological and clinical behavior. In total, high-risk neuroblastoma accounts for approximately 15% of all cancer-related deaths in this age group [3]. First-line therapy consists of induction chemotherapy, surgery, high-dose myeloablative chemotherapy followed by autologous stem cell reinfusion, radiation of the primary tumor site and remaining active metastatic lesions, as well as five cycles of the chimeric monoclonal antibody dinutuximab beta in the consolidation phase [4,5]. Further, the combination of dinutuximab beta with irinotecan and temozolomide showed notable antitumor activity in patients with refractory or relapsed high-risk neuroblastoma [6,7]. A phase I/II trial investigating the feasibility of dinutuximab beta immunotherapy after HLA (human leukocyte antigen) mismatched haploidentical stem cell transplantation demonstrated no increased risk of inducing graft-versus-host disease and suggested an antitumoral effect of the new donor-derived immune system [8].

Extravasation can present a serious complication of intravenous antineoplastic therapy. In this regard, drugs are characterized by their necrotizing effect upon extravasation. While monoclonal antibodies do not show the necrotic potential of cytotoxic drugs such as anthracyclines or vinca alkaloids, inflammatory toxicity ranging from very low (i.e., bevacizumab and trastuzumab [9,10,11]) to strong (i.e., ipilimumab [12]) can occur. While to the best of our knowledge no substance-specific protocol exists for the handling of a dinutuximab-beta extravasation, generally recommended approaches for the management of drug extravasations frequently entail ceasing infusion, aspiration and removal of the cannula under aspiration, immobilization and elevation of the affected limb, application of dry warmth or cold, dabbing with 99% dimethyl sulfoxide (DMSO), and/or infiltration with hyaluronidase [10,11].

## 2. Case Presentation

**Figure 1:**
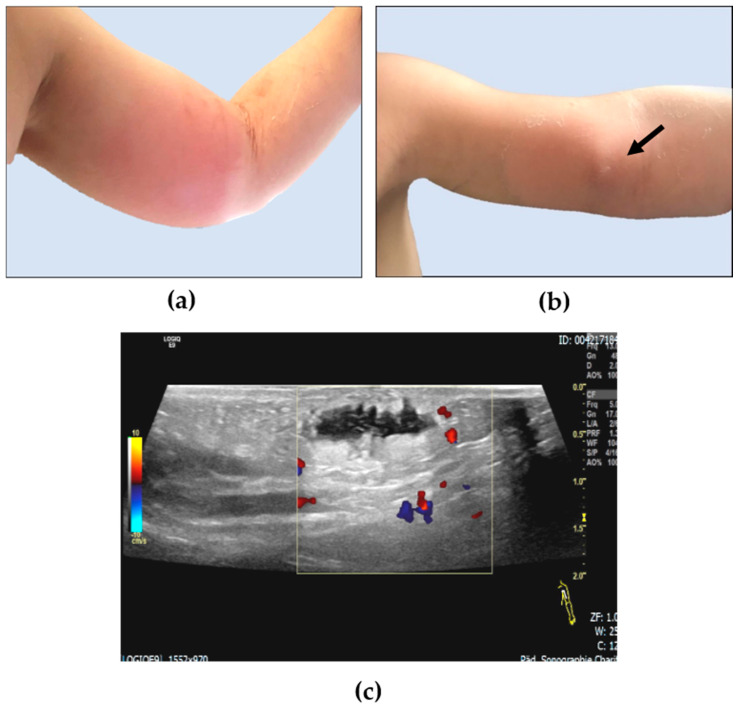
Photographic documentation of dinutuximab beta extravasation in the soft tissue of the left upper arm in a 3-year-old patient on the day of diagnosis (**a**) and on day 6 (**b**): a palpable lesion on day 6 (black arrow) was further investigated by sonography, which demonstrated an anechoic non-vascularized liquid encapsulation of 2 mL volume surrounded by panniculitis (**c**).

**Figure 2:**
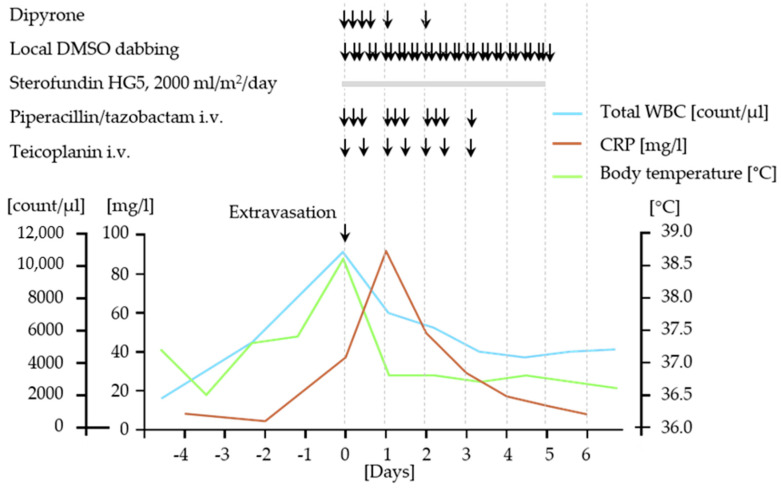
Schematic diagram illustrating the clinical symptoms, laboratory findings, and medical interventions for dinutuximab beta extravasation. DMSO, dimethyl sulfoxide; CRP, C-reactive protein; WBC, white blood count.

The 3-year-old Caucasian boy with hemophilia type B was diagnosed with *MYCN*-amplified stage M neuroblastoma at the age of 12 months and received multimodal antineoplastic therapy in line with German NB 2017 guidelines [13]. After achieving a first complete remission prior to high-dose chemotherapy followed by autologous stem cell rescue, the patient developed an isolated intracerebral/meningeal relapse after four cycles of dinutuximab beta therapy. Now, at the age of 2 years and 4 months, he was treated with three cycles of combined chemo-/immunotherapy according to Mody et al. [6,7] in combination with nine doses of intrathecal topotecan and reached a second complete remission. Following radiation of all initial macroscopically detectable lesions with 20 Gy, the patient received an allogeneic haploidentical CD19/TcRAb-depleted stem cell transplantation following a conditioning regimen composed of fludarabin, thiotepa, melphalan, and anti-T-lymphocyte globulin (ATG) via a double-lumen Hickman catheter. This catheter had to be replaced by a single-lumen Hickman catheter due to a catheter infection. The vascular situation did not allow the implantation of another double-lumen catheter. No other transplant-related complications occurred. At day 60 after haploidentical stem cell transplantation, craniospinal axis proton therapy was performed with 21.6 cobalt Gray equivalent (CGE). One month later, the first dinutuximab beta cycle was initiated in inpatient care in line with the respective protocol recommendation at 10 mg/m^2^ body weight/day as a continuous infusion over 10 days [8]. The concentration of the dinutuximab beta infusion was 0.11 mg/mL. The patient was severely immunocompromised at that time with no detectable CD3^+^, CD4^+^, and CD8^+^ cells but sufficient reconstitution of natural killer cells. The neutrophile count was 1300 per µL blood on day 1 of the cycle. The supportive treatment regimen consisted of valganciclovir, voriconazole, cotrimoxazole, ursodeoxycholic acid, and levothyroxine. Factor IX was substituted daily. On day 10 of the cycle, dislocation of the peripheral vein catheter placed in the left cubital vein alerted to dinutuximab beta extravasation (Figure 1a). The volume extravasated could not be determined with absolute certainty. The extravasated dinutuximab beta caused hyperemia and localized soft tissue swelling with a central induration of 2 × 2 cm (Figure 1a) as well as strong pain and fever (Figure 2). Laboratory diagnostics documented a white blood cell increase to a maximum of 8510 neutrophils per µl blood followed by an increase in C-reactive protein levels in the blood (Figure 2). Following immediate stop of the dinutuximab beta infusion and removal of the peripheral vein catheter under continuous aspiration, an intravenous fluid substitution at 2000 mL/m^2^/day was started to accelerate drug decomposition and elimination from the affected soft tissue. Local dabbing of the hyperemic inflamed soft tissue was carried out with 99% DMSO every four hours. Local application of dry heat was administered to denature the dinutuximab beta molecule and thereby to accelerate its decomposition. Elevated positioning of the arm was attempted but hardly tolerated by the toddler. Local infiltration with hyaluronidase was discussed but not applied due to the underlying condition of severe hemophilia type B. The patient required regular dipyrone treatment for sufficient analgesia and received a short-term intravenous treatment with antibiotics to prevent bacterial superinfection on the basis of a severely immunosuppressed host (Figure 2). The patient considerably improved after 6 days with this treatment regimen (Figure 1b). Soft tissue sonography performed at day 6 showed a residual liquid encapsulation accompanied by signs of panniculitis (Figure 1c). All symptoms and signs had fully resolved by day 20.

## 3. Discussion and Conclusions

In contrast to the absent or weak inflammatory reaction caused by extravasation of some monoclonal antibodies [9], we report here that dinutuximab beta causes a strong local and systemic inflammatory response when extravasated. The symptoms may be explained by the induction of mechanical allodynia mediated by complement activation after reactivity of dinutuximab beta with the GD2 antigen localized to the surface of peripheral nerve fibers and/or myelin in the soft tissue affected [14,15]. Both complement activation and effector cell-mediated cytotoxicity may have acutely occurred against GD2 expressed on those normal tissues as components of the soft tissue region affected. The established guidelines for handling extravasations in oncological patients were sufficient to avoid potentially irreversible tissue damage, to prevent secondary complications such as superinfections, and to reduce the clinical symptoms caused. Although all symptoms and signs were fully resolved, the prolonged recovery period of 20 days indicates that dinutuximab beta application with its generally low infusion rate must be closely monitored by experienced personnel and should, whenever possible, be conducted via a central line. Next-generation anti-GD2 antibodies with less cross-reactivity against nontumor tissue could help prevent complications such as the one reported here in the future. Candidates include complement-silenced anti-GD2 antibody variants as well as antibodies that target more tumor-specific GD2 derivatives [15,16].

## Data Availability

Not applicable.
